# Application of Outer Membrane Protein-Based Vaccines Against Major Bacterial Fish Pathogens in India

**DOI:** 10.3389/fimmu.2020.01362

**Published:** 2020-07-21

**Authors:** Biswajit Maiti, Saurabh Dubey, Hetron Mweemba Munang'andu, Iddya Karunasagar, Indrani Karunasagar, Øystein Evensen

**Affiliations:** ^1^Nitte University Centre for Science Education and Research, Mangaluru, India; ^2^Department of Paraclinical Sciences, Faculty of Veterinary Medicine, Norwegian University of Life Sciences, Oslo, Norway; ^3^NITTE (Deemed to be University), Mangaluru, India

**Keywords:** outer membrane proteins (OMPs), vaccination, fish, aquaculture, fish pathogens

## Abstract

Aquaculture is one of the fastest-growing food-producing sectors in the world. However, its growth is hampered by various disease problems due to infectious microorganisms, including Gram-negative bacteria in finfish aquaculture. Disease control in aquaculture by use of antibiotics is not recommended as it leads to antibiotic residues in the final product, selection, and spread of antibiotic resistance in the environment. Therefore, focus is on disease prevention by vaccination. All Gram-negative bacteria possess surface-associated outer membrane proteins (OMPs), some of which have long been recognized as potential vaccine candidates. OMPs are essential for maintaining the integrity and selective permeability of the bacterial membrane and play a key role in adaptive responses of bacteria such as solute and ion uptake, iron acquisition, antimicrobial resistance, serum resistance, and bile salt resistance and some adhesins have virulence attributes. Antigenic diversity among bacterial strains even within the same bacterial species has constrained vaccine developments, but OMPs that are conserved across serotypes could be used as potential candidates in vaccine development, and several studies have demonstrated their efficacy and potential as vaccine candidates. In this review, we will look into the application of OMPs for the design of vaccines based on recombinant proteins, subunit vaccines, chimeric proteins, and DNA vaccines as new-generation vaccine candidates for major bacterial pathogens of fish for sustainable aquaculture.

## Introduction

Asia accounts for more than 80% of the global aquaculture production of which India is the third largest producer (1, 2). Indian aquaculture has enormous potential and contributes significantly to the country's economy and its foreign exchange earnings. Finfishes are the most cultured species in the world, and India is no exception, contributing to 68% of world food fish aquaculture production with the other groups being molluskan shellfish (oyster, clam, mussel, and scallop), crustaceans (shrimps and prawns), and other fishes (1). A half of global food fish consumption comes from aquaculture. However, there is a need to improve management practices in order to reduce the disease burden and usage of drugs for disease treatment.

Although aquaculture is fast growing in Asian countries such as India, the largest proportion of fish farming is done by low-resource farmers in earthen ponds and floating cages in rivers and lakes whose environmental conditions support the survival of opportunistic pathogenic bacteria that cause disease in fish. Implementation of biosecurity measures is mostly low while the use of antibiotics is high, posing the danger of drug resistance (3–5). As such, vaccination is considered to be the most effective environmentally friendly disease control strategy. However, the most prevalent diseases infecting the top farmed fish species in each country guide the choice and priority of vaccine development. India being a country mostly producing carp, pathogens infecting top farmed carp species would be a priority for fish vaccines. Another confounding factor is the choice of vaccine delivery system as to whether vaccines should be administered by injection, orally, or immersion, which is guided by factors such the cost of vaccination, labor input, stress on fish, and other factors. Hence, the objective of this review is to bring into perspective the major fish species farmed in India together with major pathogens that need vaccine development. We also wanted to highlight the shortcomings of whole cell inactivated (WCI) and attenuated live vaccines used elsewhere that have paved way to research on the use of outer membrane protein (OMP) vaccines in Indian aquaculture. Herein, we provide an up-to-date status of ongoing research on OMP vaccines being developed against major pathogens infecting the top-farmed fish species in Indian aquaculture.

## Fish Aquaculture: Present Status

Food and nutritional security are being addressed through aquaculture due to stagnation of capture fisheries. In 2016, total global production of fish, crustaceans, mollusks, and other aquatic animals reached 170.9 million tons (MT) in which a large volume (>88%) was utilized for human consumption (1, 2). Global aquaculture is a fast-growing vital sector for the production of high-protein food, having an average annual growth rate of 5.8% during 2001–2016 (6). In India, aquaculture is a rapidly growing fisheries sector with an annual growth rate of over 7% of which freshwater aquaculture contributed 95% of the total annual production of 5.77 mt (MT) by 2017 (7). As discussed by Jayasankar (8), advances in carp breeding technologies and traditional polyculture system have contributed to increased production of the three India major carp species, namely, catla (*Catla catla*), rohu (*Labeo rohita*), and mrigal (*Cirrhinus mrigala*), accounting for 70–75% of total freshwater production. This is followed by the culture of three exotic carp species comprising of common carp (*Cyprinus carpio*), silver carp (*Hypophthalmichthys molitrix*), and grass carp (*Ctenopharyngodon idella*) that account for 20–30% of freshwater fish species. The increase in stocking density brought about by intensified farming systems led to increased output from 500 to 600 kg/ha to 3,000 kg/ha, resulting in fish farmers achieving higher production levels of 6–8 t/ha/year, while national average output increased from 50 kg/ha/year in 1974–1975 to about 2,135 kg/ha/year in 1994–1995 and 2,270 kg/ha/year in 2003–2004 (9, 10). Due to the contribution of public and private hatcheries in the production of about 40 billion fry in 2017, it is projected that by 2020, total carp production would exceed 15 mt due to intensive farming systems supported by high stocking densities (8). This increasing trend in stocking density could be contributing to the increase in disease outbreaks as a result of the increase in the disease transmission index as well as induction of stress predisposing fish to various infections.

## Major Bacterial Fish Pathogens in India

The major diseases of finfish in Indian aquaculture are caused by bacterial infections (7, 11). Viral pathogens like tilapia lake virus (TiLV) (12), nodavirus (13), Koi herpesvirus virus (KHV) (14), and red sea bream iridovirus (RSBIV) (15, 16) are not pathogens of top farmed fish species in India. The major parasite-causing disease in fish in India is *Ichthyophthirius mulifiliis* (11) whose impact is not severe compared to bacterial pathogens. Overall, viral and parasitic diseases cause less economic losses unlike bacterial diseases that cause an adverse economic impact, calling for the urgent need of protective vaccines (17). The most prevalent bacterial pathogens in Indian aquaculture belong to the genera *Aeromonas, Edwardsiella, Vibrio*, and *Flavobacterium*, infecting the top farmed fish species (11, 18). Table 1 shows fish species infected by these bacteria and their occurrence during different stages of the fish production cycles. Other pathogenic bacterial genera that are associated with fish diseases in India include *Streptococcus, Pseudomonas*, and *Mycobacterium*.

**Table 1:** Major bacterial diseases causing fish diseases in Indian aquaculture.

**Bacteria**	**Disease name**	**Fish spp**.	**Symptom**	**Stage of fish**	**References**
*Aeromonas hydrophila*	Motile aeromonad septicemia	Indian carp fish (catla, rohu, and mrigal)	Hemorrhagic and ulcerative lesion on the skin, fins, head	All stages	(11, 19–21)
*Edwardsiella* spp.	Edwardsiellosis	Indian carp fish (catla, rohu, and mrigal) and other cat fishes	Ulcerative abscesses in internal organs, rectal protrusion	Mostly fry and fingerlings	(11, 20–23)
*Vibrio anguillarum*	Vibriosis	Catla, rohu, mrigal, and sea bass	Hemorrhagic septicemia	All stages	(11, 20, 21)
*Flavobacterium columnare*	Columnaris disease	Catla, rohu, mrigal, common carp, and other species	Gasping, lethargic, gill looks discolored with trapped material	All stages	(11, 20, 21)

Aeromonads belong to the family *Aeromonadaceae*, and the most common species associated with fish diseases in India are *Aeromonas hydrophila* (24, 25), *Aeromonas sobria* (26, 27), *Aeromonas caviae* (28), and *Aeromonas veronii* (29). They are natural inhabitants of aquatic environments such as freshwater, estuarine, and infrequently marine waters (25). These pathogens cause hemorrhagic septicemia, tail-rot (or fin-rot), red sore, ulcerative disease, dropsy, asymptomatic septicemia, exophthalmos, and ulceration in different fish species (30).

Vibriosis is one of the most critical fish diseases caused by members of the genus *Vibrio* that are ubiquitous in aquatic environments. The disease affects both cold-water and warmwater fish species, including sea bass, carp, catfish, salmon, flounder, and eel across the world. In India, the *Vibrio* species known to cause diseases include *Vibrio anguillarum, Vibrio alginolyticus, Vibrio parahaemolyticus, Vibrio ordalii*, and *Vibrio vulnificus* of which classical vibriosis is mostly caused by *V. anguillarum* (20, 31).

Another important genus is *Edwardsiella*, which is ubiquitous in aquatic environments and is responsible for high mortality in several commercial fish species including carp, catfish, and tilapia in India. Previous studies have shown that the most common *Edwardsiella* species infecting fish in India was *Edwardsiella tarda* (22) as the causative agent of septicemia in warmwater fish species, especially catfish. It also causes fish gangrene, emphysematous putrefactive disease, red disease, and enteric septicemia in carp, catfish, and several other fish species (32, 33). However, in a recent study, we showed that piscine *Edwardsiella* isolates from 10 fish species in India belonged to *Edwardsiella piscicida* and *Edwardsiella anguillarum* (34). Therefore, it is likely that all fish isolates previously classified as *E. tarda* were either *E. piscicida* or *E. anguillarum*.

Among the *Flavobacterium, Flavobacterium columnare* is the most common isolate and often associated with columnaris in farmed catfish (Clarias batrachus), carp (*C. carpio*), rohu (*L. rohita*), catla (*C. catla*), and other fish species (35, 36). Other *Flavobacterium* species reported to cause disease in fish in India include *Flavobacterium* aquaticum, *Flavobacterium* granuli, *Flavobacterium* hercynium, and *Flavobacterium* terrae (35).

The common denominator for all these bacteria species is that they ubiquitously live in water and are able to survive under different environmental conditions (25, 37–40) becoming pathogenic as fish become vulnerable to infection when predisposing factors such as high stocking densities that stress fish leading to immunosuppression favor infection establishment. Therefore, the increase in stocking density aimed at increasing productivity in Indian aquaculture discussed in the section *Fish Aquaculture: Present Status* could be contributing to the increase in disease outbreaks caused by these bacteria species.

## Disease Prevention through Vaccination

Intensive aquaculture systems where single or multiple fish species are cultured at high densities facilitate high transmission of pathogens between individual fish. Although biosecurity measures that include quarantine, sanitation, and disinfection as well as the use of probiotics, disease-free brood stock, immunostimulants, and quality feed have been shown to reduce disease transmission, these measures do not always ensure total elimination of infectious agents. On the other hand, use of antibiotics poses the risk of selection of drug resistance in pathogens, making the treatment ineffective, spread of resistance determinants to other bacteria (41), and antibiotic residues in food (42). To prevent the recurrence of disease outbreaks and widespread use of antibiotics in aquatic environments in India, the most environment-friendly practical approach would be vaccination. For example, vaccination of Atlantic salmon (*Salmo salar* L.) against pathogens such as *Aeromonas salmonicida* and *Vibrio salmonicida* for more than 30 years contributed to a significant reduction of antibiotics use in Norway from nearly 50,000 kg of antibiotics in 1987 to <1,000–2,000 kg in 1997 (43).

Vaccines in aquaculture are either administered by injection, oral route, or immersion. Advantages of oral and immersion vaccine delivery systems are that they are less labor intensive, uses the natural route of pathogen exposure, and less stressful on fish, while vaccination by injection is labor intensive, bypasses the natural route of pathogen exposure, and is stressful on fish. However, vaccination by injection guarantees delivery of the same antigen dose to all fish, while immersion and oral vaccine delivery do not (44). Vaccines administered by injection require high labor costs for individual handling, which is expensive for the majority of low-resource fish farmers in India. On the contrary, vaccine delivery by immersion or oral does not require high labor costs because fish are vaccinated in bulk at the same time orally through feed or by immersion in vaccine-containing water. The major predicament with vaccine delivery by immersion is that practically it cannot be done in open water in ponds or cages floating in rivers and lakes. On the other hand, the major drawback with oral vaccination is that vaccines administered by ingestion are degraded in the acidic environment of the stomach/foregut before they reach the intestine where they are potentially taken up by cells of the innate immune system for local antigen presentation or transport to major immune organs (kidney/spleen) (45). There are few oral vaccines licensed to date (46), and new approaches using new technologies such as poly D, L lactic-co-glycolic acid (PLGA) nanoparticle vaccines that can protect antigens against low pH degradation in the stomach/gut are considered better alternatives.

## Choice of Vaccine Candidate: Outer Membrane Proteins

Traditionally, fish vaccines are made of live-attenuated or WCI vaccines (47, 48). WCI bacterial vaccines are prepared by chemical or heat inactivation of bacteria, and they account for the largest proportion of commercial vaccines used in aquaculture worldwide (49). They are safe because they are not infectious (“killed”) but have the disadvantage of being less immunogenic, needing adjuvants to produce long-term protective immunity (49). They elicit humoral immune responses that to a lesser extent confer protection against intracellular replicating bacteria such as *A. hydrophila, Edwardsiella* spp., or *Piscirickettsia* sp. because they do not induce cell-mediated immune (CMI) responses needed to eliminate intracellularly replicating bacteria (50). In contrast, live attenuated vaccines are highly immunogenic and have the ability to evoke both humoral and CMI responses needed to eliminate extra- and intracellular bacteria, but they pose the danger of reversion to virulence (50). DNA vaccines meet challenges of genetically modified organism (GMO) regulations; although the vaccines by themselves are not considered GMOs, vaccinated fish are considered GMOs under certain conditions. In the case of Indian aquaculture where intracellular replicating bacteria such as *A. hydrophila* and *Edwardsiella* spp. account for a large proportion of pathogens infecting top farmed fish species, there is a need for vaccines able to evoke both humoral and CMI responses. Currently, there are no licensed attenuated live vaccines against diseases caused by these pathogens. Hence, the use of genetically engineered vaccines using immunogenic proteins such as OMPs encoded in carrier vectors able to evoke both humoral and CMI responses is considered to be a better alternative.

**Figure 1:**
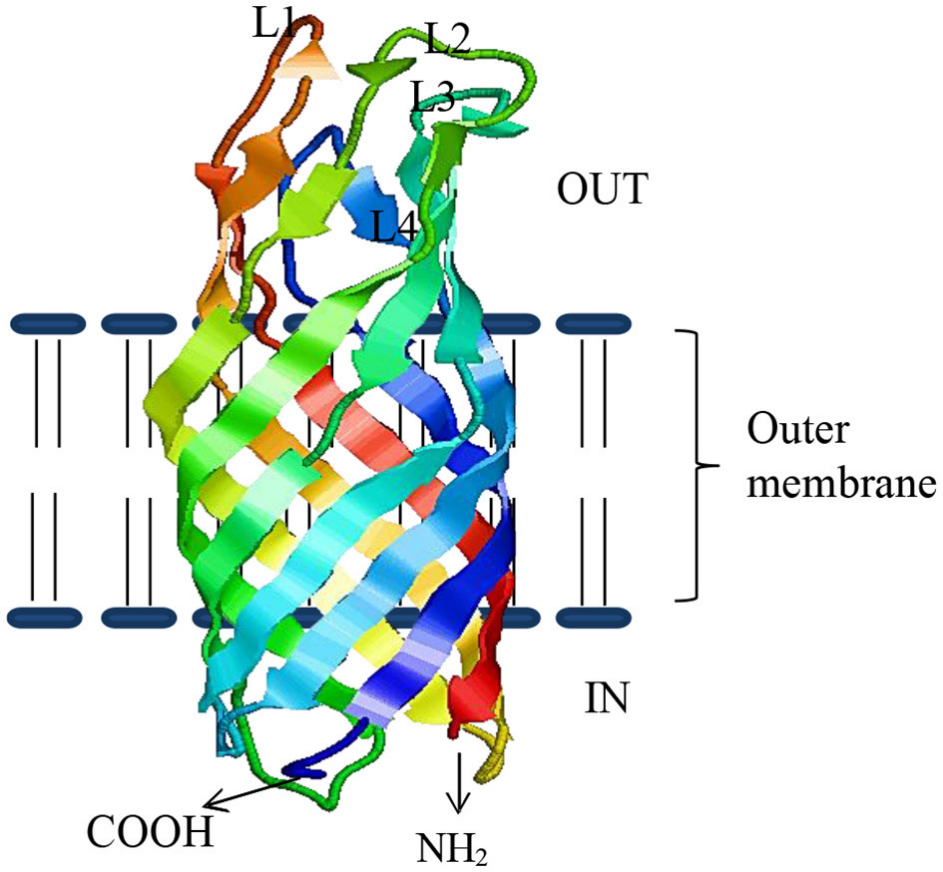
The β-sheeted architecture of OmpW protein of *A. hydrophila*. Four outside exposed loops of the protein are indicated as L1–L4, respectively.

Several studies show that bacterial OMPs have the potential to serve as vaccine candidates for immunization against bacteria infecting fish (49). OMPs are the essential component of outer membranes and are found in many prokaryotes (bacteria) as well as in specific organelles like mitochondria, chloroplasts (51) of eukaryotic cells, possibly even in archaea (52). In general, about 2–3% of the total bacterial genes encode OMPs in Gram-negative bacteria (53). They are made of β-barrel structures that contain 8–22 β-strands, which are antiparallel to each other and tilted strongly on the barrel axis (54). They are shaped in different forms such as monomers, homo-dimers, and/or homo-trimers in the outer membrane of which more than a dozen OMP structures have been resolved. As shown in Figure 1, the structural layout of OMPs shows that the C and N-terminal ends of OMPs are directed toward the periplasm, while surface loops (marked as L1, L2, L3, and so on) are located on the outermost exterior where they are exposed to the outside environment. Several studies have shown that the surface periplasmic that turns together with surface loops of OMPs have more sequence variations than the β-sheet strands, which are conserved in most bacteria species (51, 55). For example, Braun and Cole (56) found a low amino acid sequence similarity of the periplasmic turns and surface loops (54%) while β-sheets similarity was higher (74%) among OmpA proteins of *Serratia marcescens*. The strategic location of surface loops being exposed to the exterior surface render them ideal for interaction with host cells while their sequence differences could account for antigenic diversity within bacterial species (57). As such, OMPs are considered potential vaccine candidates since they are (i) highly immunogenic due to their exposed epitopes on the bacterial outer cell surface and (ii) highly conserved among different serovars and within Gram-negative bacteria (58–63). Suffice to point out that some OMPs also work as adhesins facilitating the attachment and penetration of bacteria into the host cells, thereby contributing to virulence (58–63). In addition, OMPs carry pathogen-associated molecular patterns (PAMPs) such as lipopolysaccharide (LPS) recognized by pathogen recognition receptors (PRRs) found on host cells such as monocytes, macrophages, neutrophils, and dendritic cells involved in antigen uptake, processing, and presentation to cells of the adaptive immune system for induction of long-term protective immunity. Overall, this supports the use of OMPs as ideal vaccine candidates for both intra- and extracellular bacteria that are endemic in Indian aquaculture. As shown in Table 1, various OMPs have been used for vaccine development against various pathogens infecting different fish species in India.

## Development of Vaccine Candidates through Epitope Mapping

**Figure 2:**
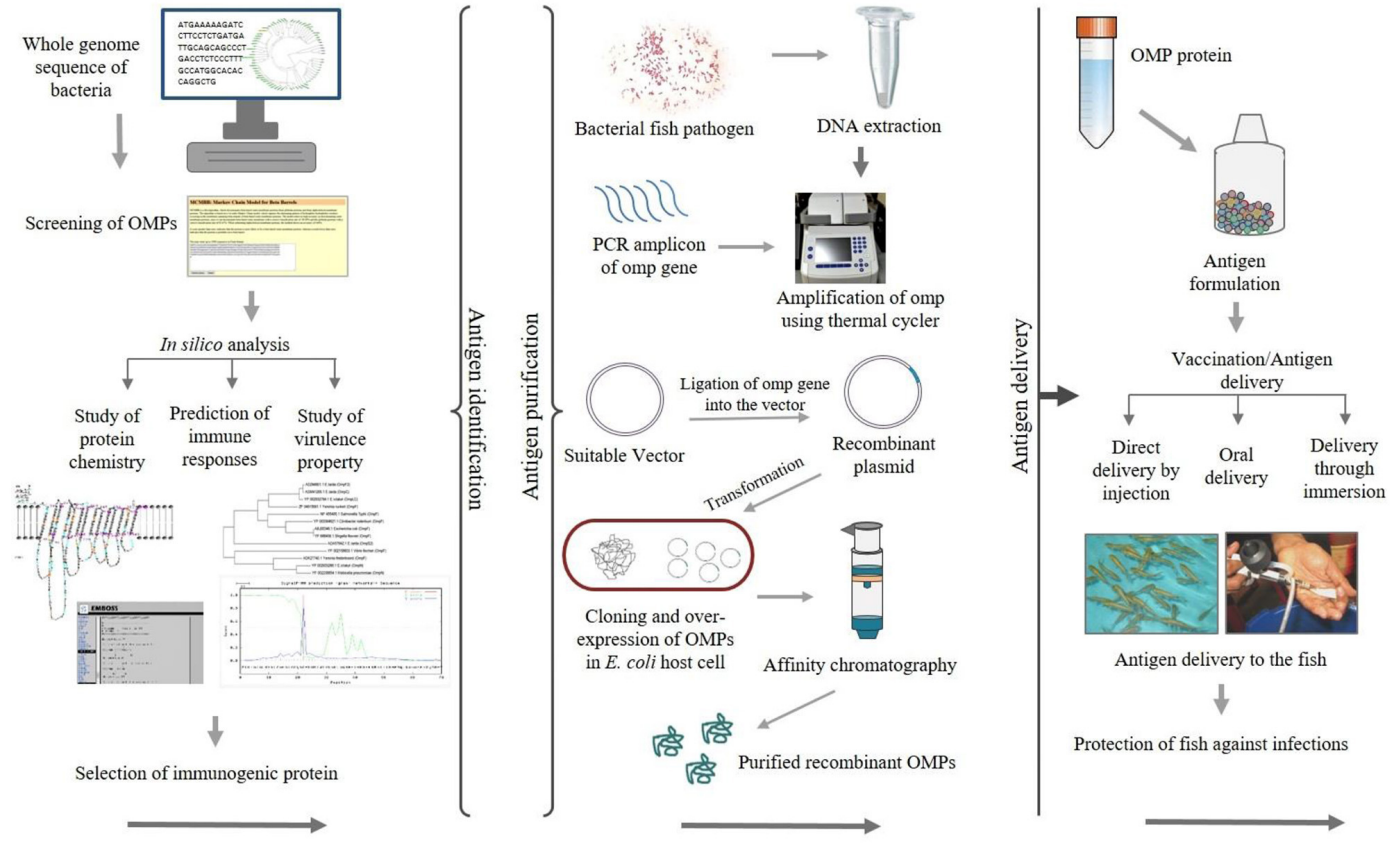
Schematic diagram showing the application of recombinant outer membrane proteins (OMPs) through a reverse vaccinology approach.

Epitope mapping of antigenic proteins recognized by B and T cells is crucial for optimal vaccine design. One approach suggested by Rappuoli (64) is to use reverse vaccinology in which several molecules are screened using *in silico* analysis to identify potential vaccine candidates (65). Figure 2 illustrates the use of reverse vaccinology in vaccine design. In India, various studies have been conducted aimed at identifying bacterial antigenic proteins using *in silico* analysis as shown in Table 2. Nucleotide or genome sequence of several OMPs can be retrieved from the databases for *in silico* analysis. There are several bioinformatics tools available used to identify open reading frames (ORFs) encoding putative *omp* genes while the basic local alignment search tool (BLAST) (http://blast.ncbi.nlm.nih.gov/Blast.cgi) of the National Center for Biotechnology Information (NCBI) can be used for sequence verification. After predicting ORFs encoding the putative *omp* genes, the next step is to apply a battery of algorithms designed to extract as much information about the ORF as possible, including tentative molecular weight, pI, and hydrophobic nature of the protein. In general, OMPs include signal peptide required for translocation from the cytoplasm to the outer membrane of cells. The SignalP 5.0 server (http://www.cbs.dtu.dk/services/SignalP/) (83) offers a platform based on a combination of several artificial neural networks that can predict the presence of signal peptides and identify the cleavage sites in proteins. The number of domains and motifs can be found with the help of a domain finder and motif finder. Further, beta-barrel OMPs can be predicted and two-dimensional topology can be analyzed using the online software PRED-TMBB (http://bioinformatics.biol.uoa.gr/PRED-TMBB) (84). The degree of immunogenicity associated with specific OMPs can be predicted using various tools such as the EMBOSS server (http://bioinfo.nhri.org.tw/gui/) (85), which is one of the popular online sites used for the determination of antigenic sites present in the protein. The presence of B- and T-cell epitopes can be identified in OMP sequences using different software. For example, the locations of linear B-cell epitopes in the OMP sequence can be identified by the BepiPred server (http://www.cbs.dtu.dk/services/BepiPred/) (86) that uses a combination of hidden Markov model and propensity scale methods. The commonly used T-cell epitope predicting tool like NetCTL (http://www.cbs.dtu.dk/services/NetCTL/) (87) identifies protein sequences using major histocompatibility complex (MHC) class I binding prediction of 12 MHC supertypes including the supertypes A26 and B39. Peptide–MHC class I binding can be predicted using NetMHC server (http://www.cbs.dtu.dk/services/NetMHC/) (88) that works on artificial neural networks (ANNs) and weight matrices.

**Table 2:** Outer membrane protein (OMP)-based vaccination studies conducted in India against major bacterial fish pathogens.

**Type of vaccine**	**Targeted OMP(s)**	**Antigen formulations**	**Bacterial species**	**Fish species**	**Method Ag delivery**	**RPS (%)**	**References**
Mixed protein	Total OMPs	With or without adjuvants	*A. hydrophila*	Goldfish (*C. auratus*)	I/p	NC (70^*^)	(66)
Mixed protein	Total OMPs	Mixed with adjuvant	*E. tarda*	Rohu (*L. rohita*)	I/p	100	(67)
Mixed protein	Total OMPs	Mixed with saline	*A. hydrophila*	Goldfish (*C. auratus*)	ND	ND	(68)
Mixed protein	Total OMPs	PLGA microparticle encapsulated	*A. hydrophila*	Rohu (*L. rohita*)	I/p	ND	(69)
Mixed protein	Total OMPs	PLA and PLGA nanoparticle encapsulated	*A. hydrophila*	Rohu (*L. rohita*)	I/p	NC (80 and 75^*^)	(70)
Mixed protein	Total OMPs	Alginate-chitosan-PLGA encapsulated	*A. hydrophila*	Rohu (*L. rohita*)	I/p	NC	(71, 72)
Subunit protein	OmpTS	Mixed with adjuvant	*A. hydrophila*	Rohu (*L. rohita*)	I/p	57	(73, 74)
Subunit protein	OmpK	Mixed with adjuvant	*V. anguillarum*	Rohu (*L. rohita*)	I/p	67.8	(75)
Subunit protein	OmpR	Mixed with adjuvant	*A. hydrophila*	Rohu (*L. rohita*)	I/p	NC	(76)
Subunit protein	Omp48	Mixed with saline	*A. hydrophila* and *E. tarda*	Rohu (*L. rohita*)	I/m	69 and 60, respectively	(77)
Subunit protein	OmpA	Mixed with saline	*E. tarda*	Common carp	I/p	54.3	(78)
Subunit protein	Aha1 and OmpW	Mixed with saline	*A. hydrophila*	Common carp	I/p	52 and 71, respectively	(58, 59)
Subunit protein	OmpW	PLGA nanoparticle encapsulated	*A. hydrophila*	Rohu (*L. rohita*)	Oral delivery	80	(79)
Subunit protein	OmpA	Chitosan nanoparticle encapsulated	*E. tarda*	Fringed-lipped peninsula carp (*L. fimbriatus*)	Oral delivery	NC (PCSP: 73)	(80)
DNA vaccine	Omp38	Chitosan microparticle encapsulated	*V. anguillarum*	Asian sea bass (*L. calcarifer*)	Oral delivery	46	(81)
DNA vaccine	Omp38	Mixed with saline	*V. anguillarum*	Sea bass (*L. calcarifer*)	I/m	55.6	(82)

* *% of survival; ND, not done; NC, not calculated*.

*RPS, relative percent survival; PCSP, post-challenge survival proportions; PLGA, poly D, L lactic-co-glycolic acid; PLA, polylactic acid*.

Another approach used for epitope mapping that has gained precedent in recent years is whole-genome sequencing (WGS) of pathogens used to identify new antigens. Together with recombinant DNA technology, WGS has contributed to improving OMP vaccine design, while protein sequence comparison has proved to be a powerful tool used to identify immunogenic proteins that are broadly protective against variant pathogen strains. For example, Dubey et al. (34) used protein sequence comparison and phylogenetic analysis to show that the OmpW of *E. piscicida* and *E. anguillarum* had high similarity, suggesting that a common antigen can be used against isolates from different fish species and geographical areas in Asia.

## Recombinant Antigen Delivery System for Outer Membrane Protein Vaccines

Genetically engineered vaccines are made of purified recombinant proteins or subunit of proteins expressed in heterologous vectors (89). The main advantage of recombinant vaccines is safety because they only contain the antigenic protein and not the entire pathogens. Moreover, genetically engineered vaccines help remove undesired harmful antigens or cleave out epitopes that stimulate T-suppressor cells. The common genetically engineered vaccines used for delivery of OMPs and other antigens in aquaculture are subunit and DNA vaccines.

### Outer Membrane Proteins as a Subunit Vaccine

In recent years, recombinant OMPs are widely tested as subunit vaccines for various pathogens in different fish species since they are highly immunogenic and they are considered safe because they only contain the antigen proteins and not the entire pathogen (59, 65, 71, 77–80, 90–105). Subunit vaccines either (i) are made of specific targeted epitopes identified from total OMPs using technologies such *in silico* analysis or mass spectrometry (Table 2) or (ii) use total OMP expressed in recombinant expression vectors (Table 3).

**Table 3:** *In silico* vaccine designing study conducted in India to control fish pathogens.

**Type of vaccine**	**Targeted OMP(s)**	**Name of bacteria pathogen**	**Conclusion from analysis**	**References**
Subunit vaccine	OmpC	*A. hydrophila*	Promising vaccine candidate	(106)
Subunit vaccine	LamB	*A. hydrophila*	A porin protein, useful as a vaccine candidate	(107)
Subunit vaccine	OmpF	*A. hydrophila*	OmpF epitope in fusion with a carrier protein, promising vaccine candidate	(108)
Subunit vaccine	TolC	*E. tarda* and *F. columnare*	Good vaccine candidate	(109)
Subunit vaccine	OmpW	*E. tarda*	An adhesin molecule, potential vaccine candidate	(63)
Subunit vaccine	OmpN	*E. ictaluri*	A porin protein, useful as a vaccine candidate	(110)
Subunit vaccine	OmpK and OmpU	*V. anguillarum*	Potential vaccine candidate	(65)

In India, Maji et al. (68) fractionated the *A. hydrophila* OMP using gel permeation and ion-exchange chromatography and generated 10 fractions of which two of the fractionated antigens made of 23-kDa and 57-kDa polypeptides had higher sero-reactivity than the crude OMP. In another study, Kumar et al. (67) used isoelectric focusing (IEF) followed by two-dimensional polyacrylamide gel electrophoresis (PAGE) and mass spectrophotometry to identify two immunogenic proteins (OMP assembly factor YaeT and GroEL) from *E. tarda* OMP that produced 100% protection after challenge in vaccinated rohu. Sharma and Dixit (106) used *in silico* analysis to eliminate nonspecific binding epitopes and selectively identified four immunodominant B-cell epitopes of the *A. hydrophila* OmpF that were highly immunogenic. They showed that the region harboring 66–80 aa residues of the OmpF had the highest reactivity in ELISA, clearly indicating that the OmpF epitope_66−80_ was the most potent vaccine candidate against *A. hydrophila*. In another study, Sharma and Dixit (108) used a bioinformatic algorithm to show that the linear B-cell epitopes covering 143–175 aa of *A. hydrophila* OmpC had the highest cross reactivity with the parent OmpC protein. Antibody isotyping, cytokine ELISA, and cytokine array analysis revealed a Th2 skewed immune response. Mahendran et al. (109) used *in silico* immunoinformatics to identify T-cell epitopes with binding interaction between *E. tarda* TolC and *F. columnare* FCOLo peptides of OMPs with MHC-I alleles. Altogether, these studies show that specific immunogenic proteins can be identified targeting B- and T-cell epitopes from total/crude OMPs for use in vaccine design.

The majority of OMP subunit vaccines are made of entire ORFs of total OMPs expressed and purified from heterologous vectors (Table 3). Bader et al. (111) showed that total OMP extracted from *Edwardsiella ictaluri* had low protection in channel catfish vaccinated with 3.13 or 6.25 μg of OMP, but a higher dose of OMP (12.5 μg) produced higher protection [relative percent survival (RPS) = 67.5]. Khushiramani et al. (73, 74) showed that the *A. hydrophila* OmpTS produced 57% survival in rohu after challenge. Similarly, Wang et al. (112) compared the protective ability of a 20-kDa protein of *A. hydrophila* OmpW with a kDa adhesin protein (Aha1) of *A. hydrophila* in common carp (*C. carpio*) and showed that the rOmpW (RPS = 71%) had superior protection over the Aha1 (RPS = 52%) after challenge (59). In another study, Khushiramani et al. (77) showed that the rOmp48 produced high protection in rohu against multiple fish pathogens viz *A. hydrophila* (RPS = 69%) and *E. tarda* (RPS = 60%), indicating that Omp48 could be used against multiple pathogens. A study by Maiti et al. (78) reported RPS = 54.3% in common carp using vaccinated rOmpA after challenge with *E. tarda*. Similarly, Hamod et al. (75) showed high protection (RPS = 67.8%) in adult rohu vaccinated with a rOmpK subunit vaccine after challenge with *Vibrio anguillarum*. Dash et al. (76) showed that a rOmpR vaccine adjuvanted with mineral oil produced 54 and 90% survival in rohu after challenge with *A. hydrophila* at 56 and 140 days post vaccination, respectively. In the same study, Dash et al. (76) used the same rOmpR vaccine with a modified adjuvant of mineral oil mixed with phosphate buffered saline (PBS) at equal volumes (1:1 ratio) and showed protection of 67 and 87% after challenge with *A. hydrophila* at 56 and 140 days post vaccination, respectively. Put together, these studies show that OMP vaccines are being developed against major fish pathogens such as *A. hydrophila, E. tarda*, and *V. anguillarum* (Table 1) and that vaccine efficacy trials are mostly done in fish species such as rohu, common carp, and channel catfish that are among the top farmed species in the Indian aquaculture. In addition, these studies also show that different OMPs such as OmpA, OmpK, OmpR, OmpW, OmpTS, and Omp48 are being used in the design of subunit vaccines in India.

### Outer Membrane Protein Encoding Genes for DNA Vaccines

Another important vaccination approach used for the delivery of OMP antigens is the use of plasmid vectors to produce DNA vaccines able to transcribe and translate the immunogenic OMP genes intracellularly (113). DNA vaccines possess several advantages over WCI vaccines such as the stimulation of both humoral and CMI responses (50, 114, 115) unlike WCI vaccines that only stimulate humoral responses (49). Moreover, DNA vaccines do not require the potentiation effect of adjuvants unlike WCI vaccines that have been shown to have severe side effects caused by adjuvants incorporated in vaccine formulations (116). In addition, DNA vaccines do not pose the danger of reversion to virulence unlike live attenuated vaccine that pose the risk of reverting to virulence. However, there are some drawbacks associated with DNA vaccination of which the most important is the possibility of integration of plasmid DNA into the host genome, which pose the danger of being transferred to other aquatic organisms and humans (117). Other difficulties include the cost of preparation and method of administration. In India, a porin gene encoding 38-kDa major OMP (Omp38) of *V. anguillarum* was used to construct a DNA vaccine for immunization of sea bass (*Lates calcarifer*) administered by intramuscular injection by Kumar et al. (82). After challenge with *V. anguillarum*, vaccinated sea bass was protected (RPS = 55.6%) unlike the control group that had high mortality. In another trial, Asian sea bass vaccinated using a DNA vaccine showed moderate protection (RPS = 46%) after challenge with *V. anguillarum* (81). Overall, there are few studies on OMP-based DNA vaccine compared to those with subunit vaccines for fish carried out in India so far.

## Biodegradable Nanoparticle Delivery Systems

PLGA, polylactic acid (PLA), and chitosan are polymers, commonly used for vaccine delivery as nanoparticles because of their biodegradable nontoxic properties (118–120). Moreover, they are easy to produce and are relatively affordable. They are attractive for oral vaccination because they easily adsorb to epithelial cells and penetrate the mucosal barrier where they are taken up by antigen-presenting cells (APCs). And as such, they can be bioengineered to enhance their adsorption on mucosal cells. Cellular uptake of PLGA nanoparticles is widely documented as shown that they are easily engulfed by various phagocytic cells such as monocytes, macrophages, neutrophils, and dendritic cells that serve as APCs leading to activation of cells of the adaptive immune system for induction of long-term protective immunity (121–126). They protect the vaccines from degradation, and they have been shown to have some potentiation effect able to enhance their uptake and enable slow release of antigens at deposition sites (127–129). Put together, these attributes render use of biodegradable nanoparticles as a better option for oral delivery of OMP vaccines than feed-coated oral vaccines.

Behera et al. (69) used PLGA microparticle for delivery *A. hydrophila* OMPs in rohu in which they observed an increase in several innate immune parameters such as respiratory burst, lysozyme, and complement activity alongside an increase in long-term expression of antibody responses against *A. hydrophila* in rohu. In another study, Behera et al. (72) showed 90% survival in rohu vaccinated with *A. hydrophila* OMP PLGA microsphere vaccine than in control fish vaccinated with OMPs that had 100% mortality after challenge with *A. hydrophila*. Similarly, Rauta and Nayak (70) showed high antibody responses and survival in rohu vaccinated with PLA-OMP (80%) and PLGA-OMP (75%) nanoparticles after challenge with *A. hydrophila*. Dubey et al. (79) immunized rohu using OmpW encapsulated in PLGA nanoparticles by oral vaccination and showed a dose-dependent protective immunity in which fish vaccinated with a low antigen dose had 48.3% survival while fish vaccinated with a high antigen dose had 73.3 % after challenge with *A. hydrophila*. In another study, Dubey et al. (80) showed that the OmpA encapsulated in chitosan nanoparticles (73.3%) had superior protection over WCI vaccine (48.3%) in *Labeo fimbriatus* after challenge with *E. tarda*. In general, studies on biodegradable nanoparticle fish vaccines are increasing in India because of safety and ease of administration orally through feed and absence of side effects. On the other hand, WCI vaccine formulations with adjuvants such as mineral oils have been linked to side effects in fish (116).

## Challenges in Developing Outer Membrane Protein Vaccines

While OMPs have proved to be protective antigens ideal for vaccine development, there are several factors that make the design of fish vaccines using OMPs a challenge. For example, the surface loops that encode epitopes for B-cell binding have been shown to be highly divergent for some bacterial species, making it difficult to choose antigens with a broad protective ability against variant strains for use in different ecosystems. One of the challenges in bioengineering of OMP vaccines is LPS detoxification. LPS activates the innate immune system via Toll-like receptor (TLR)4 of which excessive TLR4 activation causes endotoxicity, leading to excessive inflammatory cytokine expression (130). While Zollinger et al. (131) described detoxification of LPS using a detergent extraction process, other scientists have used bioengineering techniques for LPS detoxification (132, 133). Leitner et al. (134) showed that genetic modification of LPS lipid A of *Vibrio cholerae* detoxified the LPS activity and elicited the production of highly protective antibodies, while Watkins et al. (135) detoxified LPS by producing truncated LPS containing lipid IVa instead of full LPS. Endotoxicity of LPS encoded in OMPs used for fish vaccine design has not been determined, and this poses a threat in the safety of OMPs used for fish vaccines. Another challenge in OMP-based vaccines is selecting epitopes able to evoke both humoral and CMI responses. OMP surface antigens encode epitopes specific for B-cell binding (136), while luminal antigens shielded inside β-sheets have been shown to be skewed toward CMI responses (137). The challenge is to identify luminal peptides suitable for producing T-cell vaccines. While OMPs are, by themselves, potent adjuvants able to activate the innate immune system through interaction between their PAMPs and host TLRs, they require additional conventional adjuvants to sustain long-term activation of the innate immune system of which the choice of adjuvant can be a challenge especially for oral vaccines (138, 139).

## Conclusion

OMPs are essential molecules of Gram-negative bacteria as they play various roles including adaptation, immunogenicity, and pathogenesis of bacterium. They possess epitopes essential for binding to B and T lymphocytes, rendering them to be ideal vaccine candidates for both extra- and intra-cellular replicating bacteria. And as shown herein, they have been widely used in vaccine development for the Indian aquaculture, which has a high prevalence of both extra- and intracellular replicating bacterial pathogens such as *Edwardsiella* spp., *Aeromonads, Vibrio* spp., and *Flavobacterium* spp. The quest to develop safe vaccines that do not pose the danger of reversion to virulence, such as live attenuated vaccines, coupled with the need for vaccines able to evoke both humoral and CMI responses, unlike WCI vaccines, has extended the search for protective vaccines to include OMPs in vaccine development. Evidence obtained through work carried out by several groups in India reveals that OMPs are potent immunogenic molecules able to provide significant protection in fish when delivered as subunit, DNA, or PLGA/chitosan nanoparticle vaccines. Despite so, there is a need for optimization of several factors such as the choice of antigen delivery systems whether to use intra- or extra-cellular delivery as well as whether to use oral, immersion, or injectable vaccine delivery systems and to develop prime-boost vaccination regimes that confer the highest protection throughout the fish production cycle. Nonetheless, this review shows that OMP subunit, DNA, and PLGA/chitosan nanoparticle vaccines could form a large proportion of future vaccines for fish bacterial diseases in India.

## Author Contributions

BM conceptualized the initial draft of the manuscript. SD and HM conceived and reviewed the manuscript. ØE, IdK, and InK revised the manuscript. All authors read and approved publication of the manuscript.

## Conflict of Interest

The authors declare that the research was conducted in the absence of any commercial or financial relationships that could be construed as a potential conflict of interest.
